# Preventing relapse in recurrent depression using mindfulness-based cognitive therapy, antidepressant medication or the combination: trial design and protocol of the MOMENT study

**DOI:** 10.1186/1471-244X-12-125

**Published:** 2012-08-27

**Authors:** Marloes J Huijbers, Jan Spijker, A Rogier T Donders, Digna JF van Schaik, Patricia van Oppen, Henricus G Ruhé, Marc B J Blom, Willem A Nolen, Johan Ormel, Gert Jan van der Wilt, Willem Kuyken, Philip Spinhoven, Anne E M Speckens

**Affiliations:** 1 Department of Psychiatry, Radboud University Nijmegen Medical Center, Reinier Postlaan 10, Nijmegen 6525 GC, The Netherlands; 2 Pro Persona Ede, Willy Brandtlaan 20, Ede 6716 RR, The Netherlands; 3 Department of Epidemiology, Biostatistics, and Health Technology Assessment, Radboud University Nijmegen Medical Center, Geert Grooteplein 21, Nijmegen 6525 EZ, The Netherlands; 4 GGZ inGeest, partner VU University Medical Center, A.J. Ernststraat 1187, Amsterdam 1081 HL, The Netherlands; 5 Department of Psychiatry, Academic Medical Center, University of Amsterdam, Meibergdreef 5, Amsterdam 1105 AZ, The Netherlands; 6 Parnassia Bavo Psychiatric Institute, Lijnbaan 4, The Hague, 2512 VA, The Netherlands; 7 Department of Psychiatry, University Medical Center Groningen, Groningen University, Hanzeplein 1, Groningen, 9713 GZ, The Netherlands; 8 Mood Disorders Centre, School of Psychology, University of Exeter, The Queen's Drive Exeter, Devon, EX4 4QJ, UK; 9 Institute of Psychology, Leiden University, Wassenaarseweg 52, AK Leiden 2333, The Netherlands

**Keywords:** Mindfulness-based cognitive therapy, Antidepressant medication, Depression, Relapse prevention, Randomized controlled trial

## Abstract

**Background:**

Depression is a common psychiatric disorder characterized by a high rate of relapse and recurrence. The most commonly used strategy to prevent relapse/recurrence is maintenance treatment with antidepressant medication (mADM). Recently, it has been shown that Mindfulness-Based Cognitive Therapy (MBCT) is at least as effective as mADM in reducing the relapse/recurrence risk. However, it is not yet known whether combination treatment of MBCT and mADM is more effective than either of these treatments alone. Given the fact that most patients have a preference for either mADM or for MBCT, the aim of the present study is to answer the following questions. First, what is the effectiveness of MBCT in addition to mADM? Second, how large is the risk of relapse/recurrence in patients withdrawing from mADM after participating in MBCT, compared to those who continue to use mADM after MBCT?

**Methods/design:**

Two parallel-group, multi-center randomized controlled trials are conducted. Adult patients with a history of depression (3 or more episodes), currently either in full or partial remission and currently treated with mADM (6 months or longer) are recruited. In the first trial, we compare mADM on its own with mADM plus MBCT. In the second trial, we compare MBCT on its own, including tapering of mADM, with mADM plus MBCT. Follow-up assessments are administered at 3-month intervals for 15 months. Primary outcome is relapse/recurrence. Secondary outcomes are time to, duration and severity of relapse/recurrence, quality of life, personality, several process variables, and incremental cost-effectiveness ratio.

**Discussion:**

Taking into account patient preferences, this study will provide information about a) the clinical and cost-effectiveness of mADM only compared with mADM plus MBCT, in patients with a preference for mADM, and b) the clinical and cost-effectiveness of withdrawing from mADM after MBCT, compared with mADM plus MBCT, in patients with a preference for MBCT.

**Trial registration:**

ClinicalTrials.gov: NCT00928980

## Background

Major depressive disorder (MDD) is one of the most prevalent psychiatric disorders characterized by high relapse and/or recurrence rates. Relapse is defined as ‘a return of symptoms satisfying the full syndrome criteria for an episode that occurs during the period of remission, but before recovery’, where remission is a period in which the individual no longer meets syndrome criteria for the disorder and has no more than minimal symptoms, and recovery is being in remission for 6 months or longer; recurrence is ’ the appearance of a new episode of MDD occurring during recovery’ [1]. In a large prospective study, a recurrence rate of 85% was observed in outpatients with MDD during a follow-up period of 15 years [2]. Furthermore, the recurrence risk has been shown to increase with 16% after each successive episode [3]. Given the high psychological as well as social and economic burden associated with MDD, relapse/recurrence prevention is extremely important. The most commonly used strategy to prevent relapse/recurrence is maintenance treatment with antidepressant medication (mADM). International guidelines recommend that patients with recurrent MDD should continue mADM for at least two years after remission [4]. A meta-analysis showed that mADM reduces relapse/recurrence rates significantly compared to placebo (18% versus 41%) based on 31 randomized controlled trials with follow-up periods ranging from 6 to 36 months [5]. However, despite the established effectiveness of mADM as a preventive strategy, it has several disadvantages. First, many patients are unwilling to continue mADM for a longer period [6] and adherence is typically low [7]. Second, many patients experience disturbing side effects [8]. Moreover, many patients prefer psychological over pharmacological treatment [9]. Psychotherapeutic approaches also seem to have long-term beneficial effects, whereas effects of mADM cease after discontinuation [10]. To address the need for psychological interventions targeting relapse prevention, Segal, Williams and Teasdale developed Mindfulness-Based Cognitive Therapy (MBCT) [11]. The aim of MBCT is not to change or eliminate depressive symptoms, but rather to relate to them in a different way, i.e. with a more accepting, mild attitude. The rationale behind the MBCT program is based on an empirically supported, theoretical framework suggesting that patients with recurrent depression become more vulnerable to developing depression as cognitive reactivity increases (for a review see [12]). Cognitive reactivity refers to negative modes of thinking and behaving that are reactivated in periods of stress or low mood. It is suggested that these (automatic) negative reactions in turn, lead to a further lowering of mood, eventually turning into a depressive relapse/recurrence [13]. Cognitive reactivity is strongly related to rumination, which refers to recurrently thinking about one’s depressive symptoms and their possible causes and implications. Rumination is thought to be an important cognitive vulnerability factor for both onset and relapse/recurrence in depression [14,15]. MBCT is targeted at recognizing these cognitive and behavioral reactions to low mood or other stressful situations, and to observe these reactions with acceptance and kindness and from a wider, decentered perspective. Indeed, there is evidence that MBCT diminishes the ‘toxic’ relationship between post-treatment cognitive reactivity and depressive relapse [16] and that decreased rumination mediates the effects of MBCT [17]. Unlike cognitive reactivity and rumination, self-compassion seems to be a beneficial factor that is protective against depression. Self-compassion can be described as a combination of (a) self-kindness - being kind and understanding toward oneself in instances of pain or failure, (b) common humanity - perceiving one’s experiences as part of the larger human experience, and (c) mindfulness - holding painful thoughts and feelings in balanced awareness [18]. Evidence suggests that both self-compassion and mindfulness skills mediate the effect of MBCT on relapse/recurrence [16]. Three randomized controlled trials (RCTs) have shown that MBCT in addition to treatment as usual (TAU) significantly reduced the relapse/recurrence risk compared with TAU alone, over a period of 14 months [19-21]. In the first two trials [19,20], beneficial effects of MBCT were seen in patients with three or more past episodes, whereas no difference in relapse/recurrence percentages between MBCT and TAU was observed in patients with two past episodes (but see [22] for positive effects of MBCT in patients with one or two past episodes). Another trial has shown that MBCT’s prophylactic effect is at least equal to mADM for patients with three or more past episodes [23]. This latter finding may specifically apply to patients whose remission is unstable [24]. There is also evidence that MBCT might reduce subthreshold depressive symptoms, an important risk factor for relapse/recurrence, in patients remitted from MDD [22] and patients with current MDD [17]. A recent meta-analysis [25] showed that the overall risk ratio for relapse/recurrence after MBCT is 0.66 (a relative risk reduction of 34%) compared with TAU or placebo indicating that MBCT is indeed an effective prophylactic intervention for patients with recurrent MDD in remission. However, the prophylactic effectiveness of the *combination* of MBCT plus mADM has not yet been compared with either mADM or MBCT on their own. More specifically, MBCT has not been studied as an additional treatment in patients continuing mADM, rather than TAU, to prevent relapse/recurrence. Also, up to now we do not know if continuing mADM after MBCT has additional benefits over withdrawing from mADM after MBCT. As more and more MBCT courses are available, answering these specific questions becomes increasingly important for patients and clinicians in order to find the optimal strategy to prevent relapse/recurrence. The current study is designed to answer these questions.

### Aims

The purpose of the ‘MOMENT’ study is twofold using two separate but connected RCTs to answer the following questions: 1) “In patients who are in remission of depression, who are being prescribed mADM and who are reluctant to discontinue medical treatment, is MBCT when given as an add-on therapy superior to the continuation of medical treatment alone?” (mADM versus mADM + MBCT) and 2) “In patients who are in remission of depression who are being prescribed mADM and who are willing to try MBCT and not unwilling to discontinue medical treatment, is MBCT with a tapering off regimen of the medical treatment not inferior to MBCT in combination with continued medical treatment?” (MBCT versus mADM + MBCT). Our primary outcome is relapse and/or recurrence. Thus, the results of this study will inform patients with MDD and mental health professionals about the relapse and recurrence risks associated with the different treatment options given a certain treatment preference, and will support decision making processes regarding these options. In addition to these primary aims, we intend to examine; 3) the effect of mADM versus mADM + MBCT on the time to, number, duration and severity of relapse/recurrence, quality of life, and personality; 4) the effect of MBCT versus mADM + MBCT on the time to number, duration and severity of relapse/recurrence, quality of life, and personality; 5) several process variables such as MBCT adherence, rumination, cognitive reactivity, mindfulness skills, and self-compassion as possible mechanisms underlying the clinical effectiveness of MBCT; 6) the cost-effectiveness of mADM versus mADM + MBCT and 7) the cost-effectiveness of MBCT versus mADM + MBCT.

## Methods

### Design

**Figure 1:**
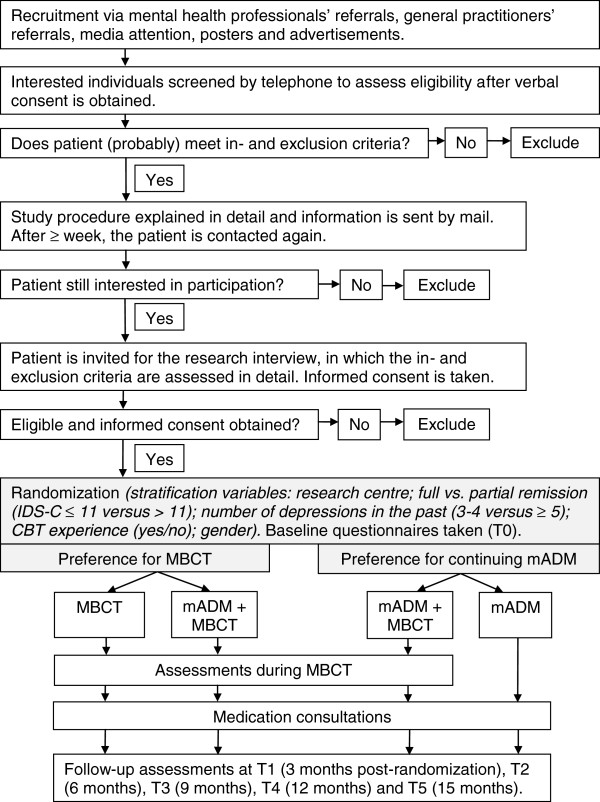
Flow chart of the recruitment and study procedure.

Originally the study was designed as a single trial, randomizing patients who are in remission of depression over [a] continuation of medical treatment, [b] switching to MBCT, or [c] MBCT as an add-on to medical treatment. This protocol was approved by our ethical review board (CMO Arnhem-Nijmegen) and registered under number 2008/242. However, inclusion of patients was hampered by the fact that many patients turned out to have a strong treatment preference: some patients were eager to start with MBCT and other patients were reluctant to discontinue medical treatment. Continuing the trial as a trial with preference arms would have resulted in a substantial proportion of patients who would not have been randomly allocated to treatment, introducing a potential serious bias. We therefore decided to conduct two separate RCTs, one with patients who are reluctant to discontinue medical treatment, and one with patients who are particularly eager to try MBCT and at least not unwilling to discontinue medical treatment. In the former, patients are randomly allocated to either continuation of medical treatment, or continuation of medical treatment with MBCT as an add-on therapy (a parallel-group, randomized controlled superiority trial). In the latter, patients are allocated to either MBCT while continuing medical treatment, or to MBCT in combination with a tapering-off regimen of the medical treatment (a parallel-group, randomized controlled non-inferiority trial). In this way, we take optimal account of the patient preferences and the study population will be as representative as possible of patients seen in routine clinical practice. The change in protocol has been approved by the Medical Ethics Committee Arnhem-Nijmegen (20-02-2011). Here, we report the design of the two trials. See Figure 1 for a flow chart of the recruitment and study procedure.

### Sample size

#### *Trial 1: mADM* versus *mADM + MBCT*

For trial 1 we need to recruit 96 participants (n = 48 per group) in order to demonstrate a difference of 25% in relapse/recurrence rates between mADM and mADM + MBCT, with a power of 80% (alpha 0.05, one-sided). This calculation is based on earlier studies reporting relapse/recurrence percentages of 60% in the mADM group [23] and approximately 38% in the MBCT plus TAU group [25]. Because our trial investigates MBCT plus mADM rather than MBCT plus TAU, we expect an even lower relapse/recurrence percentage (about 35%) in the combination group. Our expected difference is therefore 60% (mADM) minus 35% (mADM + MBCT) resulting in 25%.

#### *Trial 2: MBCT* versus *mADM + MBCT*

The sample size of trial 2 is based on the principle of non-inferiority. According to the Draft Guidance for Industry Non-Inferiority Clinical Trials of the US Food and Drug Administration [26] non-inferiority should be demonstrated by comparing the new, experimental treatment with an established efficacious treatment. In the case of relapse prevention in depression, this established treatment is mADM. However, since a head to head comparison of MBCT with mADM is complicated due to our design, we compare MBCT with the combination therapy (mADM + MBCT). We reasoned that the difference in relapse/recurrence between MBCT and mADM + MBCT should not be larger than the difference between the established treatment (mADM) and mADM + MBCT, which is expected to be approximately 25%. Therefore, we chose a non-inferiority margin of 25%. Based on this assumption, the sample size needed in this non-inferiority trial is 280 in total (n = 140 per group) with a power of 80% (alpha 0.05, one-sided).

### Participants

The study protocol has been approved by the Medical Ethics Committee Arnhem-Nijmegen (nr. 2008/242) for all participating sites. Local Ethics Committees approved local feasibility. Patients are included in the study only after written informed consent has been obtained. Participation is completely voluntary and patients can withdraw from the study and/or treatment at any time without having to give a reason for withdrawal and without consequences for their treatment options. Suspected serious adverse events are recorded and reported to the Medical Ethics Committee Arnhem-Nijmegen. Patients are recruited from nine centers across the Netherlands: Department of Psychiatry, Radboud University Nijmegen Medical Center; Department of Psychiatry, Academic Medical Center, Amsterdam; GGZ inGeest, partner VU University Medical Center, Amsterdam; Pro Persona Ede, Tiel and Arnhem; Parnassia Bavo Psychiatric Institute, The Hague; PsyQ Psycho Medical Programmes, The Hague; Leiden University Medical Center Leiden and GGZ Rivierduinen, Leiden and Lisse; GGZ Centraal, Amersfoort; and GGZ Noord-Holland-Noord, Alkmaar. Recruitment was done via referrals from mental health professionals and by media advertisements.

#### Inclusion criteria

Inclusion criteria for the MOMENT study are: a) MDD with a history of at least three depressive episodes according to the Diagnostic and Statistical Manual of Mental Disorders - 4th edition (DSM-IV [27]) using the Structured Clinical Interview for DSM disorders I (SCID-I [28]); b) treated with a stable dose of mADM over the last 6 months or longer; c) currently either in full or partial remission. Full remission is defined in our study as not currently meeting the criteria for a depressive episode assessed by the SCID and having a score of ≤ 11 on the Inventory of Depressive Symptomatology – Clinician rating (IDS-C_30_[29]). Partial remission is defined as not currently meeting the criteria for a depressive episode and having an IDS-C score > 11. The cut-off point of ≤ 11 > on the IDS-C corresponds to a Hamilton Rating Scale for Depression score of 8 which is often used as a cut-off score for remission [30]; d) native Dutch speaking.

#### Exclusion criteria

We exclude people in case of: a) bipolar disorder any primary psychotic disorder (current and previous), clinically relevant neurological or other somatic illness and/or current alcohol or drug dependency, assessed with the Mini International Neuropsychiatric Interview (MINI [31]); b) high dosage of benzodiazepines (> 2 mg Lorazepam equivalents daily); c) recent electroconvulsive therapy (< 3 months ago); d) previous MBCT/MBSR course and/or extensive meditation experience (e.g. retreats); e) current psychotherapy with a frequency of more than once per three weeks and f) visual hearing or cognitive impairments that impair the completion of self-report questionnaires and interviews.

### Interventions

#### mADM

All study participants are on a stable dose of mADM for at least 6 months prior to enrollment (inclusion criterion). In the mADM group participants continue their use of mADM during the study period of 15 months. After randomization, participants are seen by a study psychiatrist for a review of their mADM. For optimization of mADM, psychiatrists taking part in the study use a protocol based on national [32] and international [33] guidelines, made applicable for the MOMENT study by two experts in pharmacological treatment of MDD (WN and MB). Switching or augmenting medication is allowed between T0 and T1, and recommendations to manage side effects are provided. Compliance with mADM is measured prospectively during the whole study period using a daily calendar. Participants in the mADM condition are invited to take part in the MBCT training after the study period if they are interested.

#### mADM + MBCT

In the combination group participants are seen by a psychiatrist for a review of their mADM as described above, and are asked to continue their mADM during the study period. In addition, these patients are invited to take part in the MBCT training, a manualized group skills-training program [11]. MBCT is based on the protocol of Mindfulness-Based Stress Reduction (MBSR) which was developed by Jon Kabat-Zinn [34] combined with elements of Cognitive Behavioral Therapy (CBT [35]), turning it into a relapse prevention programme for patients with recurrent depression. The training consists of eight weekly sessions in a group (8 – 15 participants) with a duration of 2.5 hours, plus one day of silent practice between the 6th and 7th session. The silent day, although originally not in the MBCT program, was incorporated following the MBSR protocol [34] to give participants the opportunity to deepen their mindfulness practice. Formal meditation exercises that are part of the program are the body scan, sitting meditation, walking meditation and mindful movement. The program also encourages participants to cultivate awareness of everyday activities, such as eating or taking a shower. Cognitive techniques that are part of the program are education, monitoring and scheduling of activities, identification of negative automatic thoughts and devising a relapse prevention plan. Participants are expected to practice meditation at home for about an hour a day. In addition, participants in the MBCT conditions are invited to take part in three booster sessions every three months during the study period (around 3, 6 and 9 months after MBCT) to enhance their mindfulness practice through peer and teacher support and rehearsal of the key components of MBCT. *Delivery of MBCT and check for competence and adherence.* MBCT courses are provided at 12 different locations in the Netherlands and are led by one or two MBCT teachers per site. MBCT teachers were trained in the study protocol for MBCT during a 3-day training retreat in the beginning of the project, as well as at three subsequent training days every 6 months. Teaching sessions of each (pair of) teacher(s) are videotaped to check treatment integrity. Two tapes per teacher are randomly selected and rated by highly experienced MBCT/MBSR trainers. Competence and adherence are evaluated with the Mindfulness-Based Interventions – Teaching Assessment Criteria [36].

#### *MBCT (with tapering of mADM*)

In the ‘MBCT only’ condition participants are invited to take part in the MBCT course as described above. In addition, they are asked to taper off their mADM from session 7 of the MBCT course onwards. The protocol specifies a tapering scheme lasting 5 weeks for all common ADMs, especially addressing procedures to handle symptoms characteristic for discontinuation [37] (available on request). In case of more exceptional treatments (e.g. lithium addition) withdrawal is based on the shared opinion of the authors of the medication protocol. Clients are seen by a consultant psychiatrist for a minimum of 3 and a maximum of 12 appointments. The first three consultations are scheduled around session 1 (informing and preparing participants), session 7 (initiating withdrawal) and approximately four weeks after session 7 (evaluation of withdrawal). If more guidance is needed, additional appointments can be scheduled. Patients are reassured that they can restart ADM as soon as they suffer a relapse/recurrence, or when withdrawal proves to be unfeasible.

### Outcome measures

Table 1 presents an overview of the outcome measures and the time points of assessments.

**Table 1:** Overview of the measures and corresponding time points

**Measure**	**Target concept**	**T0**	**MBCT**	**T1**	**T2**	**T3**	**T4**	**T5**
SCID-I*	Diagnosis of (recurrent) MDD	●		●	●	●	●	●
IDS-C	Current depressive symptoms	●		●	●	●	●	●
CSRI	(Mental) health service use	●		●	●	●	●	●
MMAS	Medication adherence	●		●	●	●	●	●
RRS	Rumination	●		●				●
LEIDS	Cognitive reactivity	●		●				●
FFMQ	Mindfulness skills	●		●				●
SCS	Self-compassion	●		●				●
WHOQOL-bref	Quality of Life	●		●				●
EQ-5D	Quality of Life	●		●	●	●	●	●
NEO-PI-R	Personality	●						●
MAAS	Daily awareness/attention		●					
I-PANAS-SF	Positive and negative affect		●					
Calendar	Mindfulness and medication adherence, absence from work and health service use Monthly during 15-month study period													

Note. * Module depression (current and/or in the past).

#### Primary outcome measure

*Relapse/recurrence*. Relapse/recurrence is defined as meeting the DSM-IV criteria for a depressive episode at any moment during follow-up assessed by research assistants using the SCID-I. Our follow-up period has a duration of 15 months, therefore both relapse and recurrence can be observed. We refer to ‘relapse’ in case of a depressive episode occurring within 6 months after full remission and we refer to ‘recurrence’ in case of a depressive episode occurring after 6 months of full remission. In general, we refer to ‘relapse/recurrence’ to indicate a depressive episode within the study period. In order to prevent attrition and recall bias, the interviews (either face-to-face or by telephone) are performed at 3 months intervals (T1-T5). At any interview assistants review the previous 3 months since the previous contact. The research assistants received one full day of training to use the SCID-I. Interviews are audio taped to allow second-rating by an independent and blind assessor in cases of actual, borderline or probable relapse/recurrence. Previous studies on inter-rater reliability of the SCID-I have reported Cronbach’s alpha values between 0.61 and 0.80 [38,39].

#### Secondary outcome measures

*Time to relapse/recurrence* is calculated from baseline to first relapse or recurrence. *Number of relapses/recurrences* during the follow-up period is calculated. *Duration of relapse/recurrence* is expressed in two ways: first as the duration of the first relapse/recurrence and second, as the percentage depressed days (including multiple depressive episodes) of the total number of follow-up days. *Severity of depressive symptoms* at follow-up contacts is assessed using the Dutch version of the IDS-C [29]. The IDS-C has good psychometric qualities [40,41]. When the IDS-C is not administered during a depressive episode which falls in between assessments, the number of depressive symptoms according to the SCID-I (5 to 9) is used as a measure of severity. *Quality of life* is assessed using the 26-item self-report WHOQOL short version (WHOQOL-bref [42]) which assesses subjective quality of life in four domains: physical, psychological, social and environmental. *Personality* is measured with the NEO Personality Inventory Revised (NEO-PI-R [43]) which consists of five domains: neuroticism, extraversion, openness, altruism, and conscientiousness.

#### Process data

*Adherence to MBCT and adherence to mADM* is assessed during the entire study period using a calendar on which patients register on a daily basis their adherence to mindfulness exercises formal, informal, or none - and their adherence to mADM - full adherence, partial adherence (e.g. lower dosage than prescribed), or no adherence. This information is combined with the 4-item Morisky Medication Adherence Scale (MMAS [44]) with scores ranging from 0 (perfect adherence) to 4 (low adherence). *Rumination* is measured with the extended version of the Ruminative Response Scale (RRS-EXT [45]). The RRS-EXT enables distinction between ‘reflection’ and ‘brooding’, the former referring to a more adaptive, and the latter to a more maladaptive way of thinking about depression. *Cognitive reactivity* is assessed using the Leiden Index of Depression Sensitivity – Revised (LEIDS-R [46]). This scale consists of six subscales: hopelessness/suicidality, acceptance/coping, aggression, control/perfectionism, risk aversion, and rumination. To examine *mindfulness skills*, we administer the Dutch Five Facet Mindfulness Questionnaire (FFMQ [47]). The scale consists of 39 items divided into the subscales observing, describing, acting with awareness, nonjudging and nonreactivity. *Self- compassion* is measured with the Self Compassion Scale (SCS [48]). The SCS has 26 items measuring three concepts that are related to self-compassion: a) self-kindness versus self-judgment, b) common humanity versus isolation, and c) mindfulness versus over-identification. *Daily attention*. The Mindful Attention Awareness Scale (MAAS [49]) is administered before each MBCT session to assess mindful attention in daily life. *Positive and negative affect* is assessed before each MBCT session using the International Positive and Negative Affect Scale - Short Form (I-PANAS-SF [50]).

#### Cost-effectiveness

The cost-effectiveness evaluation is carried out from a societal perspective considering direct as well as indirect health costs. Data on health and social care utilization are collected prospectively for each individual patient using an adapted version of the Client Service Receipt Inventory (CSRI [51]). The CSRI includes production losses and family support. In addition prospective data are collected using a daily calendar on which participants register a) depression-related absence from work: full absence, partial absence or no absence, and b) any contacts with health care: the type of care and its duration. Unit cost estimates are derived from the national manual for cost prices in the health care sector [52]. Costs of reduced ability to work are estimated using the friction costs method, which results in a more realistic estimate than the human capital approach [53]. Treatment costs of MBCT are calculated using activity-based-costing methods, thus measuring actual resources (time of therapist, time of patients, facilities) used. All unit cost prices are adjusted to 2012 prices. Unit cost estimates are combined with resource utilization data to obtain a net cost per patient over the entire follow-up period. The EuroQoL-5 Dimensions instrument (EQ-5D [54]) is administered to provide utilities. The EQ-5D consists of 5 dimensions: mobility, self-care, daily activities, pain/discomfort and anxiety/depression. In addition, it contains a Visual Analogue Scale to determine Quality Adjusted Life Years (QALYs).

### Procedure

#### Assessment of eligibility informed consent and baseline assessment

Figure 1 provides an overview of the recruitment and study procedure. After informed consent is obtained eligibility is assessed during the baseline interview (T0) using the SCID I depression module, the IDS-C, and the MINI (modules bipolar disorder, psychotic disorder, alcohol and drug dependency). After randomization, the participant is informed about the condition to which he or she has been randomized. Also, the baseline questionnaires are administered at T0 (see Table 1).

#### Randomization

Randomization is computerized using a minimization strategy while stratifying over the following variables: a) research centre, b) full versus partial remission (IDS-C score ≤ 11 versus > 11), c) number of depressive episodes in the past (3-4 versus ≥ 5), d) prior CBT (yes/no) and e) gender. Sub–threshold symptoms (partial remission) and number of past episodes are stratified because both are associated with relapse/recurrence risk [55]. Also, prior CBT is stratified because this has been shown to decrease relapse/recurrence risk [56]. Randomization is performed online by the research assistant who conducts the baseline assessment by entering the required information on a randomization website specifically designed for this study. The research assistant then communicates the treatment allocation to the patient, which means that he or she is no longer blind to the treatment condition. Unblinding of patients and research-assistants could not be avoided because the different conditions required different arrangements for treatment appointments, and separating this task from the assessments was logistically impossible in most research centers. To assess the reliability of the follow-up assessments, all interviews are audio taped and a random selection of actual, borderline or probable cases of relapse/recurrence is rated by an independent assistant blind to treatment allocation.

#### Follow-up assessments

In accordance with previous trials [19,20,23], follow-up assessments take place at 3, 6, 9, 12 and 15 months post randomization (Table 1). The follow-up assessments at T2, T3 and T4 are administered by telephone and consist of only an interview part. If participants miss one or more assessments, research assistants examine the entire period from the last contact. In case of drop-out, we send a short questionnaire to gather essential information about depressive relapse/recurrence (if applicable) and the main reason for drop-out.

### Statistical analysis

#### Primary analyses

Our primary analyses will be based on intention-to-treat. Subsequently per-protocol analyses will be conducted. All analyses will be performed with and without covariates (i.e. the stratification factors research centre, depressive symptoms at baseline, and number of depressive episodes in the past, as well as other variables that might inadvertently be unevenly distributed over the conditions at baseline). *Trial 1: mADM* versus *mADM + MBCT.* The primary outcome measure will be relapse and/or recurrence meeting DSM-IV criteria for a major depressive episode during the 15-month study period. Relapse/recurrence rates will be compared with a Chi-square test. *Trial 2: MBCT* versus *mADM + MBCT.* The primary outcome measure will be relapse and/or recurrence meeting DSM-IV criteria for a major depressive episode during the 15-month study period. Relapse/recurrence rates will be compared with a General Linear Model using a binomial family with an identity link. We will use the confidence interval (one-sided) of the difference in relapse/recurrence between the two conditions (MBCT versus mADM + MBCT): if a difference of 25% can be excluded, then we will conclude non-inferiority of MBCT in comparison with mADM.

#### Secondary analyses

##### Time to relapse and number, duration and severity of relapse/recurrence

Differences in time to relapse/recurrence between mADM versus mADM + MBCT (trial 1) and between MBCT versus mADM + MBCT (trial 2) will be analyzed using a Cox Regression Proportional Hazards Model. In case of drop-out from the trial we will use the available measures and censor the participant at the time of the last assessment or informative contact. In patients suffering a relapse/recurrence during the study period we will compare the number, duration and severity of relapse/recurrence between mADM versus mADM + MBCT (trial 1) and between MBCT versus mADM + MBCT (trial 2) using a General Linear Model. We will perform additional analyses comparing different subgroups, for example patients who were in full remission at baseline (IDS-C ≤ 11) with patients who were in partial remission at baseline (IDS-C > 11) for both trials.

##### Mechanisms of change

Mediation analyses will be used to investigate the possible underlying mechanisms of change in MBCT. In accordance with other trials, these analyses will only include patients who have received an ‘adequate dose’ of MBCT, which is defined as participation in ≥ 4 of 8 MBCT sessions [16,19]. In this subsample, we will test the mediating effect of adherence to MBCT, rumination, cognitive reactivity, mindfulness skills, and self-compassion, on depressive relapse/recurrence and depression severity as outcomes, using a multiple mediation model following the approach suggested by Preacher and Hayes [57]. In addition, we will use Hierarchical Linear Modeling techniques to investigate whether change in daily attention/awareness causally influences positive and negative effect. We will perform multilevel mediational analyses following the procedure reported by Kenny, Korchmaros & Bolger [58]. We will ‘lag’ the mediator variable (daily attention/awareness) by examining whether changes on the MAAS at time *t-1* account for changes in the outcome variable (i.e. positive and negative affect) at time *t* for every MBCT session.

##### Cost-effectiveness

Cost-effectiveness of mADM versus mADM + MBCT will be analyzed in trial 1 and cost-effectiveness of MBCT versus mADM + MBCT will be analyzed in trial 2. A non-parametric bootstrapping method will be used, performing 1000 replications of the original data to produce confidence intervals. Changes in health-related quality of life from baseline will be used to calculate QALYs in each group. Incremental cost-effectiveness will be expressed in terms of incremental costs per QALY gained. A cost-acceptability curve will be constructed for statistical analysis of the incremental cost-effectiveness ratio. In case of dominance a full cost analysis will be conducted to estimate the mean savings per patient per year. To estimate the long-term consequences of introducing MBCT in the prevention of relapse/recurrence, decision analytic modeling (TreeAge) will be used, comparing mADM, MBCT, and the combination of mADM and MBCT in patients with recurrent depression, over a period of 5 years. Estimates of costs, utilities, and probabilities will be derived from the trial (extrapolation) and, where available, the literature, or from experts. Sensitivity analyses will be conducted to explore sensitivity of the outcomes to various model assumptions.

## Discussion

The prevention of relapse and recurrence in depression is considered a key target in mental health care given the high prevalence of relapse/recurrence of MDD and the accompanying (societal) costs. Previous studies have shown that MBCT significantly reduces the relapse/recurrence risk as compared to treatment as usual [25], but MBCT has not yet been studied as an additional treatment to mADM. Therefore, our first trial will inform health care professionals and patients about the relapse/recurrence risks associated with MBCT in addition to mADM compared with continuing mADM on its own. However, given the large amount of patients who prefer psychological treatment instead of ADM [9] and the difficulties that many patients have with long term use of ADM [7], it seems useful to investigate whether a relatively short, group-based course such as MBCT can help patients taper off their antidepressants. Therefore, our second trial will inform patients and clinicians about the relapse/recurrence risks associated with tapering off mADM after MBCT, compared with continuing mADM after MBCT. This trial is based on non-inferiority because we reasoned that the effectiveness of MBCT on its own (i.e. discontinuing antidepressants after MBCT) should be at least comparable to the effectiveness of mADM on its own. The original study design was an RCT comparing mADM, MBCT, and mADM + MBCT. However, because of the strong treatment preferences that patients expressed during the beginning of the recruitment phase which hampered the randomization possibilities, we adapted our design. Instead of a direct comparison between the three treatment options (three-way randomization) we now allocate the patients to different RCTs based on their preference for either mADM (allocation between mADM and mADM + MBCT) or for MBCT (allocation between MBCT and mADM + MBCT). A methodological consequence of this adaptation is that we cannot directly compare MBCT to mADM because the MBCT preference group may differ from the mADM preference group with respect to known as well as unknown variables. The advantage that comes along with this design however, is an increase in the ecological validity of both trials. In our adapted design, the included patient groups probably more closely reflect the population(s) that we are interested in, since these preferences are obviously present in the population of remitted recurrently depressed adults who use mADM. Moreover, if we had continued using the original three-way randomization procedure, we would have lost all participants who were not willing to be randomly assigned to mADM only or to MBCT only, resulting in a highly selective sample with low generalizability. In summary, more detailed knowledge about the effectiveness of MBCT in addition to mADM, and about MBCT as a possible alternative to mADM is needed. Taking into account patient preferences, the MOMENT study will address these questions to support patients and clinicians in finding the optimal strategy to prevent depressive relapse and recurrence.

## Abbreviations

CSRI: Client Service Receipt Inventory; DSM-IV: Diagnostic and Statistical Manual of Mental Disorders - 4th edition; EQ-5D: EuroQoL-5 Dimensions instrument; FFMQ: Five Facet Mindfulness Questionnaire; IDS-C: Inventory of Depressive Symptomatology – Clinician rating; I-PANAS-SF: International Positive and Negative Affect Scale Short Form; LEIDS-R: Leiden Index of Depression Sensitivity – Revised; MAAS: Mindful Attention Awareness Scale; mADM: Maintenance Antidepressant Medication; MBCT: Mindfulness-Based Cognitive Therapy; MBSR: Mindfulness-Based Stress Reduction; MDD: Major Depressive Disorder; MINI: Mini International Neuropsychiatric Interview; MMAS: Morisky Medication Adherence Scale; NEO-PI-R: NEO Personality Inventory Revised; QALY: Quality Adjusted Life Year; RCT: Randomized Controlled Trial; RRS-EXT: Extended version of the Ruminative Response Scale; SCID-I: Structured Clinical Interview for DSM Disorders-I; SCS: Self Compassion Scale; TAU: Treatment As Usual; WHOQOL: World Health Organization Quality of Life.

## Competing interests

JS received speakers’ fees from Eli Lilly, Astra Zeneca, GlaxoSmithKline, Lundbeck and Servier. HR has received a VENI- grant from the Netherlands Organization for Health Research and Development ZonMW. MB received speakers’ fees from Lundbeck. During the years 2007-2012, WN has received grants from the Netherlands Organization for Health Research and Development ZonMW, the European Union, the Stanley Medical Research Institute, Astra Zeneca, Eli Lilly, GlaxoSmithKline and Wyeth; Jo has received speakers’ fees from Astra Zeneca, Lundbeck, Pfizer and Wyeth; and has served in advisory boards for Astra Zeneca. All other authors declare that they have no competing interests.

## Authors' contributions

All authors contributed to the design of the study. AS is the principal investigator of the study. MH and AS drafted the paper, which was added to and modified by all other authors. MH, JS, DvS, PvO, HR, MB, PhS and AS were involved in recruiting participants. WN and MB provided the medication protocol for the trial. GvdW contributed specifically to the design of the cost-effectiveness evaluation and RD contributed specifically to the statistical analysis plan. All authors read and approved the final manuscript.

## Pre-publication history

The pre-publication history for this paper can be accessed here:

http://www.biomedcentral.com/1471-244X/12/125/prepub
